# Clinical and Radiological Deterioration in a Case of Creutzfeldt–Jakob Disease following SARS-CoV-2 Infection: Hints to Accelerated Age-Dependent Neurodegeneration

**DOI:** 10.3390/biomedicines9111730

**Published:** 2021-11-19

**Authors:** Dumitru Ciolac, Renata Racila, Carolina Duarte, Maria Vasilieva, Diana Manea, Nadejda Gorincioi, Alexandra Condrea, Igor Crivorucica, Eremei Zota, Daniela Efremova, Veaceslav Crivorucica, Mihail Ciocanu, Alexandru Movila, Stanislav A. Groppa

**Affiliations:** 1 Department of Neurology, Institute of Emergency Medicine, 2004 Chisinau, Moldova; dimaciolac@gmail.com (D.C.); renata.racila@gmail.com (R.R.); fbi-miv@mail.ru (M.V.); dimanea@gmail.com (D.M.); nadica_md@yahoo.com (N.G.); alexcondrea13@gmail.com (A.C.); crivorucicaigor@gmail.com (I.C.); ezotajr@gmail.com (E.Z.); daniela.efremova88@gmail.com (D.E.); crivorucica@mail.ru (V.C.); mihai.ciocanu@gmail.com (M.C.); 2 Department of Neurology, Nicolae Testemitanu State University of Medicine and Pharmacy, 2004 Chisinau, Moldova; 3 Department of Oral Science and Translational Research, College of Dental Medicine, Nova Southeastern University, Fort Lauderdale, FL 33314, USA; cduarte@nova.edu; 4 Institute of Neuro Immune Medicine, Dr. Kiran C. Patel College of Osteopathic Medicine, Nova Southeastern University, Fort Lauderdale, FL 33314, USA

**Keywords:** Creutzfeldt–Jakob disease, SARS-CoV-2, accelerated neurodegeneration, aging

## Abstract

Systemic inflammation and the host immune responses associated with certain viral infections may accelerate the rate of neurodegeneration in patients with Creutzfeldt–Jakob disease (CJD), a rare, transmissible neurodegenerative disease. However, the effects of the newly emerged SARS-CoV-2 infection on the pathogenesis of CJD are unknown. In this study, we describe the case of an elderly female patient with sporadic CJD that exhibited clinical deterioration with the emergence of seizures and radiological neurodegenerative progression following an infection with SARS-CoV-2 and severe COVID-19. Despite efforts to control the progression of the disease, a dismal outcome ensued. This report further evidences the age-dependent neurological effects of SARS-CoV-2 infection and proposes a vulnerability to CJD and increased CJD progression following COVID-19.

## 1. Introduction

Creutzfeldt–Jakob disease (CJD) is a rare, progressive, and eventually fatal neurodegenerative disorder caused by abnormally folded human prion proteins [1]. The most common form of the disease is sporadic CJD characterized by rapidly progressing dementia, myoclonus, pyramidal and extrapyramidal signs with a characteristic pattern of electroencephalography (EEG), cerebrospinal fluid (CSF) protein, and magnetic resonance imaging (MRI) abnormalities [2]. The disease’s neuropathological features of gray matter degeneration include spongiform changes, gliosis, and neuronal loss [2]. In fact, 10–15% of the prion mutations observed in CJD patients are pathogenic [3], although they have mostly been observed following surgical contamination of the central nervous system (CNS) [4]. The diagnosis of sporadic CJD relies on a combination of clinical signs and the results of diagnostic investigations (CSF 14–3–3 protein, MRI, and EEG), and classifies CJD as definite, probable, or possible [4,5]. The development and clinical application of the real-time quaking-induced conversion (RT-QuIC) assay greatly improved the pre-mortem diagnosis of CJD as it is the most sensitive and specific diagnostic test [6], and should be the first one performed in the work-up of a patient with suspected CJD. Effective therapeutics are currently unavailable; symptomatic treatment aims to alleviate patients’ myoclonus (e.g., clonazepam, valproate), anxiety/depression, pain, or seizures. The delivery of follow-up care is centered on nursing and caregiver care, acute and long-term facility care, and palliative management [7]. The prognosis of CJD is poor, as patients die most often within months or weeks (most commonly due to aspiration pneumonia), with rare cases surviving for several years [5].

Coronavirus disease (COVID-19) is an infectious disease caused by the novel severe acute respiratory syndrome coronavirus 2 (SARS-CoV-2) that quickly escalated into a pandemic after the first reported cases in China in December 2019. The SARS-CoV-2 infection can affect individuals of any age, with elderly people being particularly vulnerable, and among which high mortality rates were observed [8]. The clinical spectrum of COVID-19 varies from an asymptomatic infection to severe pneumonia with acute respiratory distress syndrome [9,10]. Typical computed tomography findings include bilateral pulmonary ground-glass opacities, sometimes with a peripheral lung distribution [10]. Besides the respiratory manifestations, a range of neurological complications associated with a SARS-CoV-2 infection, including headache, anosmia, seizures, encephalopathy, meningitis/encephalitis, myelitis, and stroke, are being increasingly reported [11,12,13]. Reverse transcriptase polymerase chain reaction (RT-PCR)-based diagnostic tests from nasopharyngeal, nasal, or oropharyngeal specimens are considered the gold standard for the detection of the current SARS-CoV-2 infection [14]. Antigen and antibody-based diagnostic tests for SARS-CoV-2 infection are less sensitive compared with the PCR-based assays [14]. Treatment approaches include the administration of antiviral drugs (e.g., remdesivir, lopinavir/ritonavir, hydroxychloroquine), anti-SARS-CoV-2 antibody products (e.g., anti-SARS-CoV-2 monoclonal antibodies, convalescent plasma), steroids, and antithrombotic therapy [9]. Several other treatment options, such as cell-based therapy and immunomodulators (e.g., interleukin (IL) 6 inhibitors), are currently under evaluation. As patients that have recovered from COVID-19 present different physical, cognitive, and neurological symptoms, long follow-up care is mandatory, including: (a) community-based rehabilitation, (b) in- and out-patient medical rehabilitation, (c) in-patient rehabilitation in skilled nursing facilities, and (d) sheltered care [15].

Inflammation and pathogen co-infection may affect the extent of neurodegenerative alterations in sporadic CJD [16]. Similarly, systemic inflammation associated with a SARS-CoV-2 infection can potentially aggravate the clinical course of sporadic CJD, as suggested in recent reports [17,18]. Herein, we present the case of an elderly female patient with sporadic CJD that deteriorated clinically and radiologically following an infection with SARS-CoV-2.

## 2. Case Report

**Figure 1:**
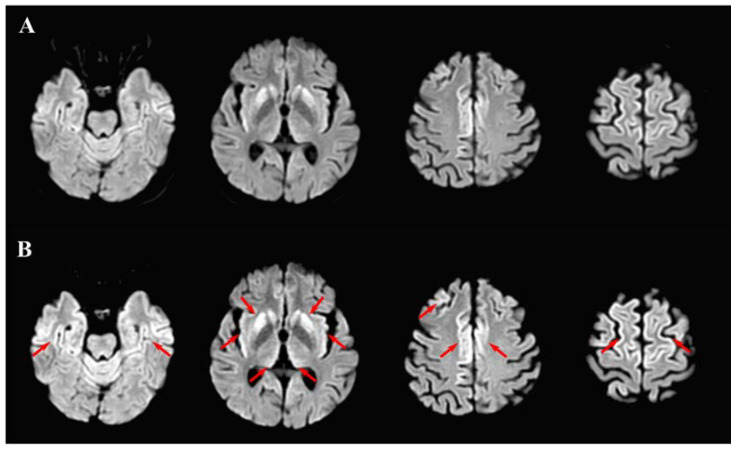
Brain MRI. Diffusion-weighted images displaying a hyperintense signal in the cortical mantle over the frontal, parietal, insular, and cingulate cortices, as well as bilateral putamina, caudate nuclei, and thalamus pulvinar before the SARS-CoV-2 infection (**A**). Repeated MRI performed nearly one month after the onset of SARS-CoV-2 infection was marked by a more enhanced signal (arrows) over the same regions (**B**).

**Figure 2:**
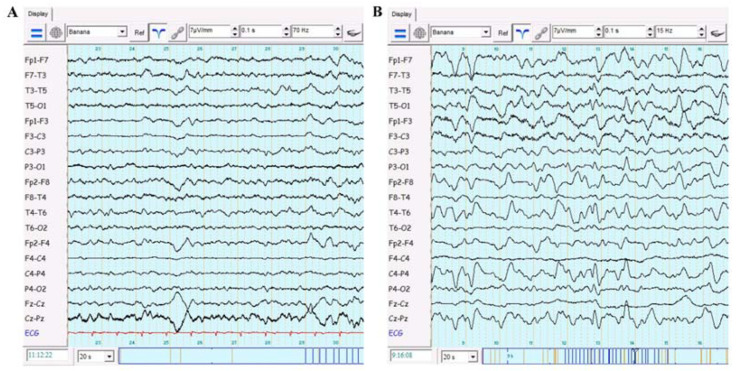
Electroencephalography (EEG). The EEG before the SARS-CoV-2 infection showing bilateral intermittent slowing (**A**). The EEG recording performed nearly one month after the onset of SARS-CoV-2 infection demonstrating bilateral quasiperiodic 1–2 Hz epileptiform discharges with biphasic and triphasic morphology (**B**).

A female patient aged in her 60s was diagnosed with sporadic CJD in December 2020 based on typical clinical, radiological, and laboratory features. Clinically, the patient presented with cognitive impairment, gait ataxia, periods of temporo-spatial disorientation, bradykinesia, and multifocal myoclonus, yet she was able to walk independently and carry out some of her daily activities. The patient’s CSF showed 14–3–3 protein, RT-QuIC assay positivity, and elevated levels of t-tau (>2000 pg/mL), f-tau (62 pg/mL), and β-amiloid (1317 pg/mL). The MRI was characterized by hyperintense signals on diffusion-weighted images (DWI) in the cortical ribbon over the frontal, parietal, insula, and cingulate cortices, as well as bilateral putamina, caudate nuclei, and thalami (Figure 1A). The EEG was dominated by periodically appearing slow activity (Figure 2A).

In January 2021, the patient presented to our hospital with headache, fever, dry cough, and shortness of breath. Her nasal and oropharyngeal swabs were positive for SARS-CoV-2 infection, and due to the severity of her symptoms, she was admitted to the COVID-19 intensive care unit (ICU). The patient’s chest computed tomography (CT) revealed bilateral basal infiltrative consolidations, while her blood analyses were unremarkable (Table 1), except for the high levels of C-reactive protein (48 mg/mL), fibrinogen (5.3 g/L), procalcitonin (0.1 ng/mL), D-dimer (1.02 mg/mL), high erythrocyte sedimentation rate (40 mm/h) (Table 2), and slightly elevated liver enzymes (Table 3). The complete blood count, inflammatory markers, and blood chemistry on the admission day and during the entire hospitalization are presented in Table 1, Table 2, Table 3.

An ECG examination revealed a sinus rhythm and left ventricular hypertrophy. Furthermore, the patient was on continuous oxygen therapy through a facial mask maintaining SpO2 levels at 94–97% and did not require mechanical ventilation. Low-dose (125 mg/day) intravenous (IV) methylprednisolone was given during the first week. The patient presented with periodic agitation and received low-dose IV dexmedetomidine or midazolam for sedation. Additionally, levetiracetam (500 mg bid) was indicated to control her myoclonic jerks. There was a gradual elevation in the number of leukocytes during her stay in COVID-19 ICU (Table 1). After a 2-week stay in the COVID-19 ICU, her respiratory symptoms and chest X-ray improved, and she was transferred to the general neurology ward. On neurological examination, mild tetraparesis, bradykinesia, bilateral cogwheel rigidity, and limb ataxia were observed. A neuropsychological examination (Montreal Cognitive Assessment test and clock-drawing test) of the patient revealed severe cognitive decline, reduced verbal fluency, poor memory and image recognition, bradyphrenia, poor executive and visuospatial function, disorientation, inattention, and apathy. Overall, a progression of neurological symptomatology occurred after a time period of almost 3 weeks after the patient was diagnosed with SARS-CoV-2 infection. A repeated 1.5T MRI examination showed a more intense signal on DWI sequences over the cortical (mainly frontal and parietal) areas and subcortical (mainly putamina and caudate) structures compared with the previous MRI scan (Figure 1B). To rule out a possible meningoencephalitis due to SARS-CoV-2 and other viral/bacterial infections, a lumbar puncture was ordered. The CSF analysis was unremarkable with normal levels of protein (0.33 g/L), glucose (4.5 mmol/L), chloride (120 mmol/L), and cell count (10/μl), and there were no traces of SARS-CoV-2 RNA. In addition, the PCR tests for Epstein–Barr virus, herpes simplex virus 1 and 2, and cytomegalovirus were negative in the CSF, and the CSF culture was negative for bacteria and fungi. The post-SARS-CoV-2 infection levels of tau proteins in the CSF were not evaluated due to in-house technical issues. Systemic inflammatory syndrome was dominated by an increased number of leukocytes and blood inflammatory markers (Table 1 and Table 2). Follow-up chest X-ray examinations showed persisting bilateral basal pneumonia with a Brixia score ranging from 2 to 4. During hospitalization, focal unaware epileptic seizures emerged, for which the dose of levetiracetam was up-titrated to a 1000 mg bid, and repeated EEG examination showed bilateral quasiperiodic epileptiform discharges (Figure 2B). The patient was unable to walk or eat independently, slowly progressing into mutism. Following a regular seizure and IV diazepam administration, the patient had respiratory depression requiring her transfer into the ICU and intubation. Unfortunately, after several days of mechanical ventilation, the patient passed away, despite efforts to resuscitate her. An autopsy examination was not performed following a decision by the patient’s relatives.

## 3. Discussion

An increasing body of evidence indicates that infection with SARS-CoV-2 can affect the CNS and can induce a range of neurological and neuropsychiatric syndromes, either by rare direct invasion or, more often, by secondary immune-mediated mechanisms [11,12,13,14,15,16,17,18,19,20]. However, less is known about the influence of SARS-CoV-2 on neurodegenerative diseases, including CJD, and on the underlying neuropathological alterations. This case report details the accelerated deterioration of a patient with pre-existing CJD after infection with SARS-CoV-2 and severe COVID-19.

As suggested in recent reports, the aggravated clinical profile of CJD in COVID-19 patients may indirectly indicate accelerated neurodegeneration following infection with SARS-CoV-2 [17,18]. The first hints arose from a case report describing a previously healthy man in his 60s who developed disorientation, an unsteady gait, non-fluent speech with phonemic paraphasia, anomia, impaired comprehension, and myoclonic jerks shortly after manifesting fever and testing positive for SARS-CoV-2 [18]. Subsequent imaging (MRI, fluorodeoxyglucose-positron emission tomography) and laboratory evaluation (CSF RT-QuIC, 14–3–3, and t-tau) confirmed a diagnosis of CJD [18], and within two months of the onset of symptoms, the patient’s clinical status progressed to mutism, right hemiplegia, multifocal myoclonus, somnolence, agitation and, eventually, death [18]. Similarly, a second case report described a previously healthy 72-year-old woman who presented with disorientation, anomia, obsessive behavior, and myoclonus following a recent COVID-19 diagnosis done retrospectively based on the patient history of anosmia, positive serum anti-SARS-CoV-2 IgG, and a chest CT scan [17]. The abnormal fluid-attenuated inversion recovery and DWI signal intensity over the bilateral parieto-occipital cortices and triphasic discharges on the EEG were signs of a sporadic CJD [17]. The patient showed no improvement after treatment and was discharged in vegetative state [17]. In addition to CJD, cases of accelerated clinical disease progression following a SARS-CoV-2 infection have been reported in the neurodegenerative disorder, Parkinson’s disease [21,22]. In the case reported here, clear neurological (including the emergence of focal seizures), EEG and MRI aggravation was observed shortly after SARS-CoV-2 infection in a patient with pre-existing CJD. Unfortunately, repeated levels of tau proteins in the CSF could not be quantified, hence definite conclusions on the underlying biochemical progression of neurodegeneration cannot be drawn. It also should be mentioned that corticosteroid therapy may be one of the factors potentially aggravating the clinical course of CJD [23]. As presented in a recent case report, the administration of corticosteroids as a pulse therapy was followed by subsequent aggravation and the occurrence of generalized myoclonus, epilepsia partialis continua, and ballistic dyskinesia in a female patient with sporadic CJD [23]. However, it is less likely that corticosteroid therapy could be the cause of deterioration in our case, as the patient received a low dose of steroids and no temporal correlation between the administration of steroids and clinical progression was observed. Based on our data and that of previous reports, it seems plausible that SARS-CoV-2 infection precipitates the clinical onset of latent neurodegenerative diseases or aggravates the evolution of an already pre-existing neurodegenerative disease.

The mechanisms by which SARS-CoV-2 influences brain neurodegenerative processes are poorly understood. It has been hypothesized that the systemic inflammation associated with a SARS-CoV-2 infection may play a critical role in the progression of neurodegeneration [24,25,26]. COVID-19 patients admitted to ICU display increased levels of inflammatory cells and pro-inflammatory cytokines (e.g., IL-1, IL-6, IL-12, interferon gamma (INF-γ), and tumor necrosis factor alpha (TNF-α) [27] that, in conjunction with local immune responses mediated by CNS-resident cells such as microglia and astrocytes, may be responsible for the precipitation and/or acceleration of prion-driven neurodegeneration [18]. Similarly in our case, the number of leukocytes began to increase in COVID-19 ICU and were particularly high at the time when neurological deterioration was noticeable. High levels of TNF-α and INF-γ, the cytokines found to correlate with viral loads in SARS-CoV-2 infection, enhance the neurotoxic effects of reactive astrocytes, which mediate neuronal damage and serve as foci for prion protein propagation [16,17,18,19,20,21,22,23,24,25,26,27,28]. Furthermore, astrocyte and microglial overactivation of cathepsins is a major contributor to neurodegeneration in sporadic CJD [29], and a recent animal study demonstrated an age-dependent increase in the genetic expression and protein activation of macrophage cathepsins in response to the SARS-CoV-2 spike protein [30]. Besides the inflammatory mechanisms of accelerated neurodegeneration, the direct neurodegenerative potential of SARS-CoV-2 can be postulated. The exposure of neurons to SARS-CoV-2 results in neuronal death due to the abnormal intracellular distribution of tau proteins and hyperphosphorylation, as demonstrated in 3D models of human brain organoids [31]. Moreover, seeded protein aggregation induced by SARS-CoV-2 was suggested as a putative mechanism of long-term post-infectious complications, including neurodegeneration [32]. However, the currently available data do not allow us to implicitly assign a direct neurodegenerative potential to SARS-CoV-2 infection, and further studies are necessary to elucidate this mechanism.

## 4. Conclusions

The SARS-CoV-2-associated systemic immune response can potentially aggravate the clinical course in patients with sporadic CJD. However, the link between the SARS-CoV-2-triggered inflammation and neurodegeneration remains elusive. Long-term data supported by pathological and biochemical evidence are required to address how the molecular pathways of SARS-CoV-2 impact on the pathogenesis of neurodegenerative disorders.

## Figures and Tables

**Table 1:** Evolution of complete blood count parameters.

Parameter	Range	COVID-19 ICU			Neurology Ward			ICU	
		Day 1 (Admission)	Day 5	Day 10	Day 1	Day 4 (Neurological Aggravation)	Day 8	Day 1	Day 3 (Death)
HGB	120–140 g/L	126	132	**116**	**114**	124	**105**	**116**	**118**
RBC	3.7–4.7 × 10^12^/L	4.1	4.7	3.9	**3.6**	4.4	**3.2**	3.9	4
PLT	150–400 × 10^9^/L	209	186	222	207	296	**119**	275	239
WBC	4–9 × 10^9^/L	4.9	**12.4**	**20.6**	**27.5**	**17.6**	**19.7**	**27.3**	**25.2**
NEUT	47–72 (%)	49	**75**	**77**	**84**	63	60	45	**23**
LY	19–37 (%)	32	**5**	**15**	**3**	22	**15**	**6**	**15**

Values with bold indicate abnormal results. COVID-19: coronavirus disease; HGB: hemoglobin; LY: lymphocytes; NEUT: neutrophils; PLT: platelets; RBC: red blood cells; ICU: intensive care unit; WBC: white blood cells.

**Table 2:** Evolution of inflammatory markers.

Parameter	Range	COVID-19 ICU			Neurology Ward			ICU	
		Day 1 (Admission)	Day 5	Day 10	Day 1	Day 4 (Neurological Aggravation)	Day 8	Day 1	Day 3 (Death)
CRP	<6 (mg/mL)	**48**	**32**	**66**	-	**46**	-	**48**	-
Fibrinogen	2–4 (g/L)	**5.3**	**5.7**	**6.9**	**6.4**	**5.3**	**4.1**	**6**	**4.8**
D-dimer	0–0.5 mg/mL	**1.02**	**1.64**	-	-	**1.8**	-	**1.72**	**-**
Procalcitonin	<0.05 ng/mL	**0.1**	**0.1**	-	-	**0.25**	**-**	**4**	**-**
ESR	2–15 mm/h	**40**	**51**	**26**	**24**	**22**	**17**	**19**	**21**

Values with bold indicate abnormal results. COVID-19: coronavirus disease; CRP: C-reactive protein; ESR: erythrocyte sedimentation rate; ICU: intensive care unit.

**Table 3:** Evolution of biochemical parameters.

Parameter	Range	COVID-19 ICU			Neurology Ward			ICU	
		Day 1 (Admission)	Day 5	Day 10	Day 1	Day 4 (Neurological Aggravation)	Day 8	Day 1	Day 3 (Death)
AST	<40 U/L	**51**	30	20	**94**	**41**	38	**42**	**44**
ALT	<41 U/L	**50**	**73**	**132**	**77**	**72**	**43**	34	39
Glucose	3.3–5.5 mmol/L	5.2	**6.9**	4.8	**7.2**	3.9	5.4	**6.3**	**6**
BUN	2.5–8.3 mmol/L	6.7	**8.5**	7.4	**16.5**	7.9	4.7	**8.8**	8.5
Creatinine	44–88 mmol/L	77	64	87	77	58	35	59	70
Protein	64–83 g/L	82	64	**52**	**48**	**52**	**47**	**50**	**52**

Values with bold indicate abnormal results. AST: aspartate aminotransferase; ALT: alanine aminotransferase; BUN: blood urea nitrogen; COVID-19: coronavirus disease; CRP: C-reactive protein; ESR: erythrocyte sedimentation rate; ICU: intensive care unit.

## Data Availability

The data presented in this study are available on request from the corresponding author. The data are not publicly available due to patient privacy concerns.
